# EARLY-LIFE EXPOSURE TO THE GREAT DEPRESSION AND THE PACE OF BIOLOGICAL AGING

**DOI:** 10.1093/geroni/igad104.0679

**Published:** 2023-12-21

**Authors:** Lauren Schmitz

**Affiliations:** University of Wisconsin-Madison, Madison, Wisconsin, United States

## Abstract

The Developmental Origins of Health and Disease hypothesis proposes that insults to early-life development can result in increased risk for aging-related disease later in life. There is growing interest within gerontology in the potential role such early life exposures play in shaping processes of aging. However, causal research linking early-life exposures to processes of aging in later life is rare. Moreover, there are difficulties in distinguishing the impacts of exposures occurring at different stages of early life development. In this study, we build on a quasi-natural experiment design we developed that leverages state-year variation in economic shocks during the Great Depression to examine the causal effect of environmental exposure in early life on aging outcomes in the US Health and Retirement Study (HRS) (2022 PNAS). Our design allows us to distinguish effects of exposure during different periods of early life development – in-utero, in the first year of life, and later in childhood. In the present study, we apply this framework specifically to study impacts of early-life exposure to the Great Depression on pace of aging in later life. We measure pace of aging using repeated-measures longitudinal functional test and biomarker data and, in parallel, a single-timepoint blood-DNA methylation assessment of the DunedinPACE epigenetic clock through the 2016 HRS assessment wave. We further examine whether impacts of the Great Depression on the pace of aging translate into differences in morbidity and mortality through 2020.

